# Sleep, 24-h activity rhythms, and plasma markers of neurodegenerative disease

**DOI:** 10.1038/s41598-020-77830-4

**Published:** 2020-11-26

**Authors:** Thom S. Lysen, M. Arfan Ikram, Mohsen Ghanbari, Annemarie I. Luik

**Affiliations:** Department of Epidemiology, Erasmus MC, University Medical Center, Doctor Molewaterplein 40, 3015 GD Rotterdam, The Netherlands

**Keywords:** Sleep, Risk factors, Dementia, Alzheimer's disease, Neural ageing

## Abstract

Sleep and 24-h activity rhythm disturbances are associated with development of neurodegenerative diseases and related pathophysiological processes in the brain. We determined the cross-sectional relation of sleep and 24-h activity rhythm disturbances with plasma-based biomarkers that might signal neurodegenerative disease, in 4712 middle-aged and elderly non-demented persons. Sleep and activity rhythms were measured using the Pittsburgh Sleep Quality Index and actigraphy. Simoa assays were used to measure plasma levels of neurofilament light chain, and additionally β-amyloid 40, β-amyloid 42, and total-tau. We used linear regression, adjusting for relevant confounders, and corrected for multiple testing. We found no associations of self-rated sleep, actigraphy-estimated sleep and 24-h activity rhythms with neurofilament light chain after confounder adjustment and correction for multiple testing, except for a non-linear association of self-rated time in bed with neurofilament light chain (*P* = 2.5*10^−4^). Similarly, we observed no significant associations with β-amyloid 40, β-amyloid 42, and total-tau after multiple testing correction. We conclude that sleep and 24-h activity rhythm disturbances were not consistently associated with neuronal damage as indicated by plasma neurofilament light chain in this population-based sample middle-aged and elderly non-demented persons. Further studies are needed to determine the associations of sleep and 24-h activity rhythm disturbances with NfL-related neuronal damage.

## Introduction

Sleep and 24-h activity rhythm disturbances have been implicated in the etiology of clinical neurodegenerative diseases and several pathophysiological processes in the brain^1–4^. The habitual level of disturbed sleep and 24-h activity rhythms, as a repetitive exposure of potential insults to the brain over a prolonged period of time^1,3^, may be particularly important for pathophysiological processes in the brain. Although most studies on the effect of sleep and 24-h activity rhythm disturbances have focused on β-amyloid and tau pathology, central hallmarks of Alzheimer’s disease^1,3,5^, plasma markers of neuronal damage have been studied less^5^.

Neuronal damage can be captured in vivo by cerebrospinal fluid levels of the cytoskeletal protein neurofilament light chain (NfL)^6,7^. Importantly, NfL can also be determined less invasively in blood^8^, higher levels of which may mark neurodegenerative diseases and other neurological disorders^9–11^. Moreover, NfL in blood has been shown to consistently rise at a higher rate years before the diagnosis in persons who eventually develop Alzheimer’s disease versus healthy controls^10,12^. This suggests NfL can be used as a biomarker of clinically relevant pathophysiological processes in the brain, even though NfL in blood may lag behind neuronal damage, e.g. one month after an isolated neurosurgical trauma^13^. Additionally, at a cellular level, NfL release may signal neuro-axonal damage that does not necessarily lead to neuronal loss^14,15^. This suggest that NfL may be of added value to existing studies using non-invasive neuroimaging markers as it may detect more subtle damage to the brain^5,16,17^.

Several studies used NfL to determine the relation of sleep, but not yet 24-h activity rhythms, with neuronal damage^18–25^. One study showed that persons with chronic insomnia disorder have higher serum NfL than controls, which may decrease after treatment^18^. Others found no relation of disordered, subjectively impaired or experimentally deprived sleep with NfL in CSF or plasma^19–25^. No large-scale population-based study has yet investigated associations of objectively estimated habitual sleep and 24-h activity rhythm disturbances with neuronal damage indicated by NfL, even though this repetitive exposure of disturbed sleep might impact neuronal damage.

We therefore tested the association of habitual sleep and 24-h activity rhythms with neuronal damage in middle-aged and elderly non-demented individuals from the population-based Rotterdam Study cohort. We assessed characteristics of habitual sleep and 24-h activity rhythms with a self-reported questionnaire indicating sleep quality over the past month and one week of actigraphy, which estimates sleep and 24-h activity rhythms objectively based on wrist movement. These modalities have been suggested to tap into different aspects of habitual sleep^26^. We assessed NfL in plasma to indicate neuronal damage. We hypothesized that self-rated and actigraphy-estimated poor sleep, and disturbed 24-h activity rhythms were associated with higher plasma NfL. For comparison, we also studied associations of sleep and 24-h activity rhythms with other plasma biomarkers of pathophysiological processes in the brain (β-amyloid 40 [Aβ_40_], Aβ_42_, and total tau [t-tau]).

## Methods

### Study setting

This study is embedded in the population-based, prospective Rotterdam Study cohort, which includes individuals from a suburban district in Rotterdam, the Netherlands^27^. The cohort was initiated in 1990, including 7983 participants aged ≥ 55 years, and was expanded in 2000 with 3011 participants aged ≥ 55 years, and again in 2006 with persons aged ≥ 45 years, totaling 14,926 participants. Examination rounds include a home interview and subsequent visits to our dedicated research center, and are repeated every 4 to 5 years.

The Rotterdam Study has been approved by the medical ethics committee of the Erasmus MC (registration number MEC 02.1015) according to the Population Screening Act, executed by the Ministry of Health, Welfare and Sports of the Netherlands. The study was performed in accordance with the Declaration of Helsinki. Written informed consent was obtained from all participants.

### Study population

Between 2002 and 2005, 6044 participants from the initiation and first expansion cohort underwent venipuncture at the research center. Of those, 5069 had sufficient plasma stores available for analyzing biomarkers. We excluded 232 persons without valid data on plasma NfL, and 20 persons with all-cause dementia to focus on at-risk individuals only. From the remaining 4817 participants, 4712 provided valid data on ≥ 1 questionnaire-derived sleep parameter (4353 persons provided data on all parameters).

Also, out of aforementioned 4817 participants, 1346 individuals were invited to participate in an actigraphy study^28^; 970 agreed. Of these, 849 persons (88%) provided valid data for a minimum of 4 consecutive 24-h periods^28^.

### Self-rated sleep

Participants rated their sleep using a Dutch version of the Pittsburgh Sleep Quality Index (PSQI^29^). The PSQI measures sleep over the past month, and has good test–retest reliability and validity in a non-clinical sample of older adults. Items include bedtimes and total sleep time at night, from which we derived time in bed and sleep efficiency, and time to fall asleep (sleep latency). Additionally, all items were summed to obtain the global PSQI score, indicating subjective sleep quality. The PSQI score ranges from 0–21, and higher scores indicate a poorer subjective sleep quality.

We excluded persons missing ≥ 2 PSQI components (n = 60), and calculated a weighted global PSQI score when only 1 component was missing (n = 173) by multiplying the six-component sum score by 7/6. The PSQI was completed a median of 18 days (interquartile range [IQR] = 17–19) before venipuncture.

### Objectively estimated sleep and 24-h activity rhythms

Participants wore an actigraph (Actiwatch model AW4, Cambridge Technology Ltd.) which measures acceleration summed as ‘activity counts’ per 30-s epochs. We instructed participants to wear the actigraph for 7 days and nights around the non-dominant wrist, and to remove it only while bathing. Participants had to press a marker button on the device when attempting to fall asleep (hereafter: ‘lights out’), and when getting out of bed the next morning (hereafter: ‘lights on’). They also kept a daily sleep diary^28^. Missing marker times (25%) were imputed from the diary, or estimated by inspecting recordings if diary times were missing. We removed 24-h periods containing > 3 continuous hours without activity to prevent bias from removal of data around specific times of the day. Actigraphy recordings averaged 137.9 ± 13.6 h, and were initiated a median of 28 days (IQR 9–287) after venipuncture. Within the marker-defined time in bed, we estimated sleep (i.e. total sleep time) and wakefulness using a validated algorithm with a threshold of 20 counts^28^. We defined ‘sleep start’ as the midpoint of the first immobile ≥ 10 min period after ‘lights out’ with ≤ 1 epoch containing activity^28^. Sleep onset latency was calculated as the time from ‘lights out’ to ‘sleep start’, and wake after sleep onset as wakefulness after ‘sleep start’. We calculated sleep efficiency as total sleep time divided by time in bed * 100%.

We also used counts to calculate non-parametric indices of the 24-h activity rhythm: Intradaily variability which indicates the amount of alterations of activity-inactivity, interdaily stability which indicates how daily profiles in the recording resemble each other, and onset time of the least active 5 consecutive hours (L5 onset) which indicates the phase of lowest activity. A disturbed 24-h activity rhythm is reflected by high intradaily variability and interdaily stability.

### *Measurement of plasma concentrations of NfL, Aβ*_*40*_*, Aβ*_*42*_*, and t-tau*

Participants came to the dedicated research center where a venipuncture was performed between 8:00 and 10:30 in the morning after an overnight fast. Blood was sampled in ethylenediamine tetra-acetic acid-treated containers and centrifuged. The plasma was aliquoted and frozen at − 80 °C according to standard procedures. In 2018, samples were assessed through the Janssen Prevention Center (Leiden, NL) which sent plasma to the laboratory facilities of Quanterix (Lexington, MA, USA). Analyses were performed on a single molecule array (Simoa) HD-1 analyzer platform in two batches^30^. Concentrations of biomarkers were measured using the NF-light advantage kit^31^ (for NfL), and the Simoa Human Neurology 3-Plex A assay (for Aβ_40_, Aβ_42_, and t-tau). Samples were tested in duplicate, two quality control samples were run on each plate per biomarker. Technical data on assay performance was published previously^10^. Data was excluded if duplicates or single measurements were missing, if the concentration coefficient of variation exceeded 20%, or if control samples were out of range.

### Covariates

As potential confounders we selected possible causes of the determinant or the outcome, or proxies of such factors^32–34^, in line with recent literature^35^. We considered age, sex, education (categorized as primary, secondary/lower vocational, intermediate vocational and higher vocational/university), batch number of biomarker analysis, time interval between measurements of sleep and biomarker, habitual alcohol consumption, presence of self-reported paid employment, smoking status (never, former, current), body mass index (BMI), presence of hypertension (resting blood pressure > 140/90 mmHg, or use of blood pressure-lowering medication), presence of diabetes mellitus (fasting serum glucose level ≥ 7.0 mmol/l, or use of glucose-lowering medication), total cholesterol level in serum in mmol/l, a positive history of heart disease (myocardial infarction, heart failure, or coronary revascularization procedure), and possible sleep apnea defined using PSQI items on loud snoring and respiratory pauses. Measurements were performed during the home interview or center visits, as detailed previously^36^.

Additionally, we assessed clinically relevant depressive symptoms defined as a score < 16 on the validated Dutch version of the Centre for Epidemiological Studies-Depression scale (CES-D), cognitive impairment defined by a Mini Mental State Examination (MMSE) score ≤ 25, and a history of stroke ascertained during examination rounds and by continuous monitoring as detailed previously.

### Statistical analysis

All sleep parameters were winsorized at 3 SD from the mean, and subsequently standardized. Biomarker values were log-transformed (base = 2) to approach a normal distribution, winsorized to 3 SD and standardized to facilitate comparison across different biomarkers.

We used linear regression to analyze the association of sleep and 24-h activity rhythm parameters with plasma NfL. We investigated self-rated sleep (PSQI score, total sleep time, sleep onset latency, time in bed, and sleep efficiency), actigraphy-estimated sleep (total sleep time, sleep onset latency, wake after sleep onset, time in bed, sleep efficiency), 24-h activity rhythms (intradaily variability, interdaily stability and L5 onset) and times of ‘lights out’ and ‘lights on’. Analyses were adjusted for age, sex, educational level, batch, and time interval between measurements of sleep and biomarkers (model 1), and additionally for alcohol consumption, paid employment status, smoking status, BMI, hypertension, diabetes mellitus, total cholesterol, history of heart disease, and possible sleep apnea (model 2). Furthermore, as total sleep time and time in bed are known to show U-shaped relations with various poor health outcomes, we assessed non-linear associations of these parameters (self-rated and actigraphy-estimated) with NfL by adding their quadratic terms to the model.

We additionally restricted analyses to persons without clinically relevant depressive symptoms, without cognitive impairment, and without prevalent stroke. Depressive symptoms may strongly influence sleep and sleep’s appraisal^37^, and depression is associated with cortical abnormalities^38^. Cognitive impairment is a proxy for the accumulation of detrimental processes in the brain potentially influencing the relation of sleep or 24-h activity rhythms with neurodegeneration, and may influence reporting of sleep. Likewise, prevalent stroke is a proxy for higher loads of cerebrovascular disease potentially affecting NfL and sleep^33,34^.

Besides NfL, other biomarkers may also be potentially important. Therefore, we also examined associations of sleep and 24-h activity rhythms with other plasma biomarkers of neurodegenerative disease: Aβ_40_, Aβ_42_, and t-tau.

We performed statistical testing with two-tailed tests, and considered associations below the threshold of *P* < 0.0046 as statistically significant, which corrected for testing 15 self-rated and actigraphy-estimated parameters in this study. This threshold was defined by computing the number of effective tests (M_eff_ = 11.14) based on correlations between all parameters, and applying a Sidak correction. We considered associations as nominally significant at *P* < 0.05.

Missing values on covariates were imputed using five multiple imputations with IBM SPSS Statistics version 24 (IBM Corp, Armonk, NY). Analyses were performed with R software^39^.

## Results

We included 4712 participants (mean age 72 ± 8 years, 57% female), see Table 1. The median plasma NfL level was 13.3 pg/mL (IQR 10.0–18.4).

**Table 1:** Characteristics of study population.

Characteristic (unit)	Total sample	Actigraphy subsample
	N = 4712	N = 849
Age at sleep measurement (years)	71.1 (66.1–77.2)	66.7 (63.7–73.1)
Female	2700 (57%)	433 (51%)
**Educational level**		
Primary education	536 (11%)	53 (6%)
Lower/intermediate or lower vocational	2088 (44%)	368 (43%)
Higher or intermediate vocational	1436 (31%)	281 (33%)
Higher vocational or university	652 (14%)	147 (18%)
Alcohol consumption (g/day)	7 (1–20)	9 (1–20)
Paid employment	303 (6%)	74 (9%)
**Smoking status**		
Never	1480 (31%)	264 (31%)
Former	2598 (55%)	477 (56%)
Current	634 (13%)	108 (13%)
Body mass index (kg/m^2^)	27.6 ± 4.1	27.9 ± 4.0
Hypertension	2569 (54%)	414 (49%)
Diabetes mellitus	472 (10%)	84 (10%)
Total cholesterol in serum (mmol/l)	5.6 ± 1.0	5.7 ± 1.0
History of heart disease	704 (15%)	89 (10%)
Possible sleep apnea	580 (12%)	113 (13%)
**Self-rated sleep**		
Global PSQI score	3 (2–6)	3 (1–6)
Duration (h)	6.8 ± 1.3	6.9 ± 1.2
Latency (min)	10 (5–30)	10 (5–30)
Time in bed (h)	7.7 ± 1.1	7.7 ± 1.0
Efficiency (%)	93 (83–99)	93 (86–100)
**Actigraphic sleep and 24 h activity rhythms**		
Total sleep time (h)	–	6.5 ± 0.8
Latency (min)	–	18 (12–26)
Wake after sleep onset (h)	–	1.1 (0.9–1.4)
Time in bed (h)^a^	–	8.3 ± 0.8
Efficiency (%)	–	79 (74–83)
Intradaily variability (score)	–	0.41 (0.34–0.52)
Interdaily stability (score)	–	0.83 (0.76–0.88)
L5 onset (hh:mm)	–	01:53 ± 01:10
‘Lights out’ time (hh:mm)^a^	–	23:51 ± 00:48
‘Lights on’ time (hh:mm)^a^	–	08:10 ± 00:45
Neurofilament light chain (pg/ml)	13 (10–18)	11 (9–15)
Range	3–390	4–214

Characteristics of the study population. Values are expressed as frequency (%) for categorical variables and mean ± standard deviation or median (1st quartile–3rd quartile) for continuous variables. Includes imputed values for covariates. Missing values for self-rated sleep parameters were 60 for PSQI score, 58 for sleep duration, 198 for sleep latency, 159 for time in bed, and 212 for sleep efficiency.

^a^Actigraphic time in bed was not automatically calculated but based on ‘lights out’ and ’lights on’ times specified daily by participants using the actigraph marker buttons and a sleep diary.

*L5* least active 5 h of the day, *N* sample size, *PSQI* Pittsburgh Sleep Quality Index.

### Sleep parameters

For self-rated sleep parameters, we found no significant linear associations with plasma NfL in model 2 (Table 2). The association of self-rated longer time in bed with higher NfL in model 1 (beta per standard deviation [SD] increase in self-rated time in bed of 0.038 SD increase in log_2_(NfL), 95% confidence interval [CI] 0.015; 0.060, *P* = 0.0013) attenuated after additional adjustment (Table 2). The quadratic term of self-rated time in bed was significantly associated with NfL in model 2 (*P* = 2.5*10^−4^). Compared to a self-rated normal time in bed (7–9 h), spending a long time in bed (> 9 h) was significantly associated with higher NfL (0.171, 95% CI 0.086; 0.256, *P* = 7.7*10^−5^), but spending a short time in bed (< 7 h) was not (− 0.008, 95% CI − 0.057; 0.041, *P* = 0.75).

**Table 2:** Associations of self-rated and actigraphy-estimated sleep parameters with neurofilament light chain levels in plasma.

Independent variables	Model 1		Model 2	
	Beta (95% CI)	*P*	Beta (95% CI)	*P*
**Self-rated**				
PSQI score	0.023 (− 0.001; 0.046)	0.06	0.014 (− 0.009; 0.036)	0.23
Sleep duration	0.005 (− 0.018; 0.027)	0.68	0.007 (− 0.015; 0.028)	0.56
Sleep latency	0.017 (− 0.010; 0.044)	0.23	0.006 (− 0.020; 0.032)	0.65
Time in bed	0.038 (0.015; 0.060)	0.001	0.032 (0.009; 0.054)	0.01
Sleep efficiency	− 0.032 (− 0.056; − 0.008)	0.01	− 0.025 (− 0.048; − 0.001)	0.04
**Actigraphy-estimated**				
Total sleep time	− 0.006 (− 0.058; 0.047)	0.83	− 0.030 (− 0.082; 0.022)	0.26
Sleep latency	− 0.008 (− 0.062; 0.045)	0.76	− 0.004 (− 0.057; 0.048)	0.88
Wake after sleep onset	0.023 (− 0.028; 0.073)	0.37	0.021 (− 0.028; 0.071)	0.40
Time in bed^a^	0.001 (− 0.051; 0.053)	0.97	− 0.021 (− 0.073; 0.030)	0.41
Sleep efficiency	− 0.004 (− 0.055; 0.047)	0.87	− 0.016 (− 0.066; 0.034)	0.52

Estimates represent that, with a standard deviation increase in the independent variable, the level of neurofilament light chain (NfL) increases by beta*standard deviation (SD) log_2_ pg/mL. Estimates were obtained with linear regression, adjusted for age and sex, educational level, batch, time interval between measurement of sleep and biomarkers (model 1), and additionally for alcohol consumption, employment status, smoking status, body mass index, presence of hypertension, presence of diabetes mellitus, total serum cholesterol level, history of cardiovascular disease, and possible sleep apnea (model 2). Analyses were performed in n = 4652 persons for PSQI score, in n = 4654 for sleep duration, in n = 4514 for sleep latency, in n = 4552 for time in bed, and in n = 4499 for sleep efficiency. Actigraphy analyses were performed in 849 persons.

^a^Please note that actigraphy-derived time in bed was not automatically calculated but based on ‘lights out’ and ‘lights on’ times, specified daily by participants using actigraph marker buttons and a sleep diary.

*CI* confidence interval, *PSQI* Pittsburgh Sleep Quality Index.

Actigraphy-estimated sleep parameters were not related to NfL in plasma (Table 2). We found no non-linear associations for actigraphy-estimated total sleep time and time in bed.

### 24-h activity rhythm parameters

We observed no significant associations of 24-h activity rhythm parameters with NfL beyond the multiple testing corrected threshold (Table 3).

**Table 3:** Associations of actigraphy-estimated 24-h activity rhythm parameters and bedtimes with neurofilament light chain in plasma.

Independent variables	Model 1		Model 2	
	Beta (95% CI)	*P*	Beta (95% CI)	*P*
Intradaily variability	0.022 (− 0.033; 0.078)	0.43	0.036 (− 0.019; 0.092)	0.19
Interdaily stability	0.000 (− 0.051; 0.052)	0.99	− 0.017 (− 0.068; 0.033)	0.50
L5 onset	− 0.008 (− 0.059; 0.043)	0.76	− 0.005 (− 0.054; 0.045)	0.85
‘Lights out’ time^a^	− 0.050 (− 0.103; 0.004)	0.07	− 0.033 (− 0.086; 0.020)	0.22
‘Lights on’ time^a^	− 0.044 (− 0.095; 0.008)	0.10	− 0.049 (− 0.100; 0.001)	0.06

Estimates represent that, with a standard deviation increase in the independent variable, the level of neurofilament light chain (NfL) increases by beta*standard deviation (SD) log_2_ pg/mL. Estimates were obtained with linear regression, adjusted for age and sex, educational level, batch, time interval between measurement of sleep and biomarkers (model 1), and additionally for alcohol consumption, employment status, smoking status, body mass index, presence of hypertension, presence of diabetes mellitus, total serum cholesterol level, history of cardiovascular disease, and possible sleep apnea (model 2). Analyses were all performed in 849 persons.

^a^Please note that actigraphy-derived bedtimes were specified daily by participants using actigraph marker buttons and a sleep diary.

*CI* confidence interval, *L5* average least active 5 h of the day, *SD* standard deviation.

### Sensitivity analysis

Restricting the main analysis to individuals without clinically relevant depressive symptoms, without cognitive impairment or stroke overall did not substantially change effect sizes (Table 4). For self-rated time in bed, estimates were attenuated after excluding persons with cognitive impairment, and to a lesser extent after excluding persons with stroke, but not after excluding those with clinically relevant depressive symptoms (Table 4).

**Table 4:** Associations of sleep with neurofilament light chain in plasma in persons without depressive symptoms, cognitive impairment or stroke.

Independent variables	No depressive symptoms	No cognitive impairment	No stroke
	Beta (95% CI)	Beta (95% CI)	Beta (95% CI)
**Self-rated**			
PSQI	− 0.001 (− 0.026; 0.024)	0.007 (− 0.017; 0.032)	0.010 (− 0.013; 0.033)
TST	0.015 (− 0.009; 0.039)	0.001 (− 0.023; 0.025)	0.006 (− 0.016; 0.028)
SOL	0.001 (− 0.029; 0.030)	− 0.001 (− 0.029; 0.028)	0.001 (− 0.026; 0.028)
TIB	0.036 (0.012; 0.059)^†^	0.022 (− 0.002; 0.046)	0.028 (0.006; 0.051)*
SE	− 0.017 (− 0.042; 0.009)	− 0.022 (− 0.047; 0.003)	− 0.022 (− 0.046; 0.001)
**Actigraphy**			
TST	− 0.024 (− 0.077; 0.030)	− 0.027 (− 0.085; 0.030)	− 0.029 (− 0.082; 0.025)
SOL	− 0.013 (− 0.069; 0.042)	− 0.003 (− 0.061; 0.054)	− 0.003 (− 0.055; 0.049)
WASO	0.011 (− 0.040; 0.062)	0.016 (− 0.037; 0.069)	0.021 (− 0.029; 0.070)
TIB^‡^	− 0.032 (− 0.086; 0.021)	− 0.029 (− 0.085; 0.028)	− 0.016 (− 0.069; 0.036)
SE	− 0.001 (− 0.053; 0.051)	− 0.010 (− 0.064; 0.044)	− 0.016 (− 0.066; 0.034)
IV	0.018 (− 0.039; 0.076)	0.046 (− 0.015; 0.107)	0.042 (− 0.012; 0.097)
IS	− 0.006 (− 0.058; 0.047)	− 0.018 (− 0.074; 0.038)	− 0.010 (− 0.060; 0.041)
L5 onset	− 0.014 (− 0.065; 0.037)	− 0.023 (− 0.076; 0.031)	0.003 (− 0.047; 0.053)
Lights out^‡^	− 0.035 (− 0.089; 0.020)	− 0.029 (− 0.086; 0.027)	− 0.039 (− 0.091; 0.014)
Lights on^a^	− 0.063 (− 0.115; − 0.010)*	− 0.055 (− 0.111; 0.001)	− 0.050 (− 0.101; 0.001)

Absence of depressive symptoms was defined as CES-D score ≥ 16; absence of cognitive impairment was defined as MMSE score > 25.

Estimates represent that, with a standard deviation increase in the independent variable, the level of neurofilament light chain (NfL) increases by beta*standard deviation (SD) log_2_ pg/mL. Estimates were obtained with linear regression, adjusted for age and sex, educational level, batch, time interval between measurement of sleep and biomarkers, alcohol consumption, employment status, smoking status, body mass index, presence of hypertension, presence of diabetes mellitus, total serum cholesterol level, history of cardiovascular disease, and possible sleep apnea. For self-rated independent variables, cases per analysis ranged from 4048 to 4181 restricted to persons without depressive symptoms, from 3908 to 4042 in persons without cognitive impairment, and from 4288 to 4431 in persons without prevalent stroke. For actigraphy-derived independent variables, cases in analyses were n = 785 (depressive symptoms), n = 756 (cognitive impairment) and n = 817 (stroke).

*Nominal significance at *P* < 0.05.

^†^*P* = 0.0034.

^a^Actigraphic time in bed was not automatically calculated but determined by ‘lights out’ and ’lights on’ times specified through pressing actigraph marker buttons and the sleep diary.

*CES-D* Center for Epidemiological Studies-Depression scale, *CI* confidence interval, *IS* interdaily stability, *IV* intradaily variability, *L5* average least active 5 h of the day, *MMSE* mini-mental state examination, *PSQI* Pittsburgh Sleep Quality Index, *SD* standard deviation, *SE* sleep efficiency, *SOL* sleep onset latency, *TIB* time in bed, *TST* total sleep time, *WASO* wake after sleep onset.

### Associations with plasma Aβ-isoforms and total tau

For comparison, we also investigated associations of sleep and 24-h activity rhythm parameters with other biomarkers of neurodegenerative disease. Median (IQR) plasma levels in pg/mL for 4712 persons were 259.5 (230.3–294.0) for Aβ_40_, 10.3 (8.8–11.9) for Aβ_42_, and 2.4 (1.9–3.0) for t-tau. In comparison to associations with NfL, we observed more associations exceeding *P* < 0.05 including associations of poorer subjective sleep quality, longer self-rated time in bed and lower self-rated sleep efficiency with higher plasma concentrations of β-amyloid isoforms (Table 5). Yet, no association was statistically significant beyond the threshold corrected for multiple testing (Table 5).

**Table 5:** Associations of sleep and 24-h activity rhythms with biomarkers of neurodegenerative disease in plasma.

Independent variables	β-amyloid 40	β-amyloid 42	Total tau
	Beta (95% CI)	Beta (95% CI)	Beta (95% CI)
**Self-rated**			
PSQI score	0.020 (− 0.008; 0.047)	0.030 (0.002; 0.057)*	− 0.016 (− 0.045; 0.013)
TST	0.005 (− 0.021; 0.032)	− 0.007 (− 0.034; 0.020)	0.018 (− 0.009; 0.046)
SOL	0.008 (− 0.025; 0.040)	0.009 (− 0.023; 0.041)	− 0.002 (− 0.036; 0.031)
TIB	0.033 (0.006; 0.060)*	0.032 (0.005; 0.059)*	0.019 (− 0.009; 0.047)
SE	− 0.020 (− 0.048; 0.008)	− 0.038 (− 0.066; − 0.010)*	0.008 (− 0.021; 0.038)
**Actigraphy**			
TST	− 0.051 (− 0.116; 0.013)	− 0.025 (− 0.086; 0.036)	0.034 (− 0.032; 0.100)
SOL	− 0.001 (− 0.066; 0.064)	0.019 (− 0.042; 0.080)	0.007 (− 0.060; 0.074)
WASO	0.049 (− 0.012; 0.110)	0.051 (− 0.006; 0.109)	0.045 (− 0.018; 0.108)
TIB^a^	− 0.036 (− 0.099; 0.028)	0.005 (− 0.055; 0.065)	0.061 (− 0.004; 0.127)
SE	− 0.047 (− 0.109; 0.015)	− 0.050 (− 0.108; 0.008)	− 0.028 (− 0.092; 0.036)
IV	0.066 (− 0.002; 0.134)	− 0.002 (− 0.067; 0.062)	− 0.007 (− 0.077; 0.063)
IS	− 0.022 (− 0.085; 0.041)	0.018 (− 0.041; 0.077)	− 0.025 (− 0.089; 0.040)
L5 onset	0.019 (− 0.042; 0.080)	0.027 (− 0.031; 0.085)	0.020 (− 0.043; 0.083)
Lights out^a^	0.007 (− 0.058; 0.072)	− 0.017 (− 0.079; 0.044)	0.019 (− 0.048; 0.085)
Lights on^a^	− 0.027 (− 0.089; 0.036)	− 0.017 (− 0.076; 0.042)	0.074 (0.010; 0.139)*

Estimates represent that, with a standard deviation increase in the independent variable, the level of neurofilament light chain (NfL) increases by beta*standard deviation (SD) log_2_ pg/mL. Estimates were obtained with linear regression, adjusted for age and sex, educational level, batch, time interval between measurement of sleep and biomarkers, alcohol consumption, employment status, smoking status, body mass index, presence of hypertension, presence of diabetes mellitus, total serum cholesterol level, history of cardiovascular disease, and possible sleep apnea. Numbers of cases per analysis differed as both independent variables and outcomes had different numbers of missing values. For self-rated independent variables, numbers varied from 4486 (association total sleep time with total tau) to 4145 (sleep efficiency with β-amyloid 42). For actigraphy-derived independent variables (all n = 849), numbers varied from 824 (total tau) to 806 (β-amyloid 42).

*Nominal significance at *P* < 0.05.

^a^Actigraphic time in bed was not automatically calculated but based on ‘lights out’ and ’lights on’ times specified by participants.

*CI* confidence interval, *IS* interdaily stability, *IV* intradaily variability, *L5* average least active 5 h of the day, *PSQI* Pittsburgh Sleep Quality Index, *SD* standard deviation, *SE* sleep efficiency, *SOL* sleep onset latency, *TIB* time in bed, *TST* total sleep time, *WASO* wake after sleep onset.

## Discussion

In this population-based study in middle-aged and elderly persons, sleep and 24-h activity rhythms were not associated with plasma NfL, except for a non-linear association of self-rated time in bed with NfL.

We only found a non-linear association of self-rated time in bed with NfL. This is in line with findings that more sedentary behavior, although distinct from sleep, is linked to various poor health outcomes which may impact neuronal damage^40^. We might speculate that the association of self-rated long time in bed with higher plasma NfL could be due to a shared common cause such as overall poor health or underlying subclinical disease^41,42^. Indeed, the linear association of self-rated time in bed with NfL is attenuated after additional adjustment in model 2 and when persons with cognitive impairment or stroke, but not depressive symptoms, were excluded. This suggests that poor physical health or clinical diseases could underlie the association of time in bed with NfL. Other potential factors underlying the link of longer time in bed with poor health outcomes may be fatigue, immune function, or sleep apnea^42^. Further research may consider investigating if self-rated time in bed indeed validly marks poor health, and how it relates to other sleep-related markers^41^. Of note, this association was only present when time in bed was assessed through general retrospective ratings of bedtimes over the last month, but not when time in bed was based on averages obtained from prospectively collected marker buttons or daily sleep diaries. This might be explained by a difference in operationalization: the PSQI assesses time in bed independent of whether a person tries to sleep when in bed whereas this is taken into account in the assessments with actigraphy and sleep diary. Additionally, as retrospective questionnaire ratings tend to suffer more from recall bias than prospective measurements, it might also be that the association is driven by factors related to recall bias rather than time in bed per se, such as cognitive impairment.

Recently, we demonstrated that actigraphy-estimated poor sleep was associated with the risk of clinical all-cause dementia and Alzheimer’s disease in the Rotterdam Study. Yet, sleep and 24-h activity rhythm disturbances are not clearly associated with NfL in the current study which is embedded in the same cohort, suggesting that poor sleep does not affect neuronal damage as indicated by NfL. Our finding partly contradicts findings from other studies implementing non-invasive structural neuroimaging which do suggest that poor sleep and 24-h activity rhythms are related to global or regional loss of tissue or integrity^5,16,17^. Together, these and our findings suggest a role for non-neuronal, i.e. glial cells in the relation between sleep and pathology of the brain. A methodological explanation may be reverse causation; brain changes picked up by imaging affect sleep, while higher levels of plasma NfL may not necessarily indicate enough damage to the brain to affect sleep or activity rhythms. Of note, our findings are in line with previous studies that show that neuroimaging markers and NfL are correlated in the presence of neurodegenerative disease^9,43^, but show little to no correlations in otherwise healthy individuals^43–45^. Possibly, NfL may reflect brain pathology on imaging only once a certain threshold is exceeded, a suggestion also highlighted by a recent study^46^.

The lack of an association of sleep and 24-h activity rhythms with NfL could be explained in several ways. First, habitually disturbed sleep and 24-h activity rhythms may affect neuronal health yet do not lead to NfL release. At a cellular level, release of NfL, most abundantly present in the axon, occurs after apoptosis or axon-specific neuronal insults^14,15^. Sleep or 24-h activity rhythm disturbances may involve neuronal insults that, through invoking various stress responses, impair neuronal function but do not lead to apoptosis. Second, we measured sleep with questionnaires and actigraphy. These measurements may not have captured relevant sleep disorders such as insomnia or sleep-disordered breathing, or physiological aspects of sleep such as slow-wave activity. This could explain why a previous study showed higher serum NfL in persons with chronic insomnia versus controls^18^, while we found no association of subjective sleep quality, an insomnia-related construct, with NfL in the general population. Third, neuronal insults related to sleep and 24-h activity rhythm disturbances may not be severe enough to elevate NfL in plasma. Our hypothesis on the detrimental effects of poor sleep for neuronal health was based on mechanistic, animal-based studies that mostly used experimental sleep deprivation. However, we studied observational differences in habitual sleep, and these more chronic disturbances might be associated with less harm to neuronal health than experimentally induced reductions in sleep. Indeed, a previous study also did not find an association of observational differences in subjective sleep quality with NfL, using CSF measurements^20^. Additionally, experimentally depriving individuals of sleep to 4 h for five nights did not affect NfL in CSF^19^ or blood^22^. One night of total sleep deprivation also did not change NfL in blood^24^.

Compared to NfL, associations of sleep and 24-h activity rhythms with Aβ_40_, Aβ_42_ and t-tau in plasma were largely similar. Estimates suggested nominally significant relations for self-rated time in bed, and the related variable of sleep efficiency, comparable to findings for NfL. Yet, no associations survived multiple testing correction. These null findings for β-amyloid were in contrast to our expectations as sleep has been shown to regulate brain β-amyloid levels^47^, and habitual sleep has been associated with CSF β-amyloid, and parenchymal β-amyloid deposition^5^. Also, lower plasma Aβ_42_ was associated with a higher risk of Alzheimer’s disease in our cohort^10^. We measured Aβ_42_ in plasma which may differ or be less precise than measurements in CSF^48^, thus potentially obscuring detection of an association.

For tau, we could also not confirm findings of previous studies linking disturbed or disordered sleep to tau brain pathology^23,49,50^ or total tau in blood^51^, which may in part be explained by the use of polysomnography-derived characteristics of sleep in these studies.

Several methodological considerations need to be mentioned. First, our largely negative findings could indicate that our biomarker measurements, using plasma instead of CSF, were invalid. Yet, high NfL and reduced Aβ_42_ in plasma have been associated with risk of clinical all-cause dementia and Alzheimer’s disease in non-demented individuals in our cohort^10^, suggesting they may reflect neurodegenerative disease in a preclinical phase. Second, correlations of NfL between CSF and plasma are lower in healthy versus diseased persons, lowering our sensitivity to detect relevant plasma NfL increases, especially in the actigraphy subgroup. Third, associations with plasma NfL may not reflect increased damage but differential equilibration across fluid compartments, as poor sleep may disturb blood–brain barrier function. Fourth, cross-sectional associations may not have been detected as plasma NfL levels may lag behind neuronal injury, e.g. on average one month after an isolated neurosurgical trauma^13^. Yet, our single sleep measures are relatively stable over time, as are plasma NfL levels across years in relation to neurodegenerative diseases^10,43^, and we adjusted analyses for the time interval between measurements. Additionally, our cross-sectional design prevents us from speculating on the temporality of any associations. Fifth, actigraphy estimates may misclassify sleep and only indirectly reflect circadian functioning. Sixth, we could not investigate the influence of physical activity on our estimates, as the Actiwatch model used in this study was not suited for quantifying physical activity. Study strengths include using a large sample anchored in the general population, measuring sleep with two modalities, simultaneously investigating multiple relevant biomarkers, and correcting for various confounders.

In conclusion, our findings do not indicate a consistent relation of sleep and 24-h activity rhythm disturbances with plasma NfL in our population-based sample of middle-aged and elderly non-demented persons. Additionally, sleep and 24-h activity rhythm disturbances seemed also unrelated to Aβ_40,_ Aβ_42_ and t-tau in plasma. Further studies across different populations are needed to determine whether sleep and 24-h activity rhythms disturbances are not associated with neuronal damage assessed with plasma NfL.

## Data Availability

Data can be obtained on request. Requests should be directed toward the management team of the Rotterdam Study (secretariat.epi@erasmusmc.nl), which has a protocol for approving data requests. Because of restrictions based on privacy regulations and informed consent of the participants, data cannot be made freely available in a public repository.
